# PGE_2_ Modulates Uterine Luminal Fluid Composition and Endometrial Function in Dairy Heifers During Diestrus

**DOI:** 10.3390/ani16071037

**Published:** 2026-03-28

**Authors:** Beibei Zhang, Yutong Yan, Yuan Han, Longgang Yan, Dong Zhou, Pengfei Lin, Yaping Jin

**Affiliations:** 1 College of Animal Science and Technology, Fujian Vocational College of Agriculture, Fuzhou 350303, China; zbb20210610@163.com; 2 Key Laboratory of Animal Biotechnology of the Ministry of Agriculture, College of Veterinary Medicine, Northwest A&F University, Yangling 712100, China; 2021065023@nwafu.edu.cn (Y.H.); yanlonggang@nwafu.edu.cn (L.Y.); zhoudong1949@nwafu.edu.cn (D.Z.); linpengfei@nwafu.edu.cn (P.L.); 3 College of Animal Sciences, Fujian Agriculture and Forestry University, Fuzhou 350002, China; yanyutong@fafu.edu.cn

**Keywords:** dairy heifers, uterine luminal fluid, prostaglandin E_2_, lipid metabolism, endometrial function

## Abstract

Prostaglandin E_2_ (PGE_2_) plays a critical role in regulating uterine endometrial function and supporting embryonic development during early pregnancy in ruminants. However, its precise roles in shaping the uterine microenvironment remain unclear. Herein, 1 mg PGE_2_ was infused daily into the uterus of dairy heifers from days 12 to 14 of the estrus cycle. ULF was subsequently collected for integrated proteomic, metabolomic, and targeted lipidomic analyses. In addition, bovine endometrial epithelial cells were used to evaluate the effects of PGE_2_ on epithelial adhesion and responsiveness to interferon tau (IFNT). PGE_2_ infusion resulted in 909 differentially abundant proteins (DAPs), which are primarily associated with early embryonic development, immune regulation, and cell adhesion. Untargeted metabolomics analysis identified 587 altered metabolites, which were enriched in sphingolipid, arachidonic acid, phenylalanine, and tryptophan metabolism. Proteomic–metabolomic analyses showed that these alterations were primarily associated with early embryonic development, immune regulation, and cell adhesion. Targeted lipidomic analysis showed a global reduction in lipid accumulation, with glycerophospholipid metabolism and choline metabolism most significantly affected. In vitro, PGE_2_ reduced epithelial microvilli density, increased osteopontin (OPN) expression, and decreased the expression of junctional proteins (zona occludens-1 (ZO-1), E-cadherin (CDH1), and fibronectin 1 (FN1)). Moreover, PGE_2_ enhanced the responsiveness of bEECs to IFNT by interferon alpha/beta receptor 1 (IFNAR1) and IFNAR2, and prostaglandin E receptor 4 (PTGER4) was identified as the primary receptor mediating this response. Collectively, these findings suggest that PGE_2_ may modulate lipid metabolism and adhesion-related processes in the endometrium and influence endometrial responsiveness to IFNT, providing insights into molecular mechanisms associated with pregnancy establishment in dairy cows.

## Simple Summary

Prostaglandin E_2_ (PGE_2_) is a naturally occurring compound in the uterus that plays a critical role in early reproductive processes of dairy cows. However, its effects on the uterine environment remain poorly defined. In this study, PGE_2_ was administered via intrauterine infusion in dairy heifers to evaluate its impact on the composition of uterine luminal fluid (ULF) and the functional properties of uterine epithelial cells. PGE_2_ markedly altered the proteomic and metabolomic profiles of ULF, with lipid metabolism identified as the most prominently affected biological process. Furthermore, PGE_2_ modified the epithelial surface structure, upregulated cell adhesion–related proteins, and enhanced the responsiveness of epithelium cells to interferon tau (IFNT). Collectively, these findings provide insight into PGE_2_-mediated regulation of uterine function and highlight its potential role in modulating the uterine environment during pregnancy establishment in dairy cows.

## 1. Introduction

Early embryonic loss represents a major constraint on reproductive efficiency in dairy cows, with a substantial proportion of embryos failing to survive during the peri-implantation period. This phenomenon is primarily attributed to disturbances in the uterine microenvironment, particularly alterations in the composition of uterine luminal fluid (ULF) [1,2,3]. In ruminants, the hatched blastocyst undergoes a dramatic morphological transformation, elongating from an ovoid structure into an approximately 25 cm filamentous conceptus, and this process is essential for the establishment of pregnancy [4]. During the peri-implantation period, the developing conceptus is highly dependent on ULF due to its primary source of nutrients, signaling molecules, and metabolic support [5,6,7,8]. Therefore, a comprehensive understanding of ULF composition and the mechanisms regulating its formation is critical for improving pregnancy establishment and reproductive efficiency in dairy cows.

ULF, also known as histotroph, is a complex and dynamic secretion containing proteins, lipids, amino acids, ions, glucose, growth factors, hormones, enzymes, extracellular vesicles, and other bioactive molecules. These components are synthesized by the endometrial glandular epithelium (GE) and luminal epithelium (LE), selectively transported from blood, and partially generated through semi-autonomous metabolic processes supported by enzymatic activity within the uterine lumen [7,9]. Current evidence supports the concept that the uterine endometrium undergoes coordinated changes in gene expression and function during the peri-implantation period. These changes are primarily regulated by ovarian progesterone (P_4_) and signaling molecules, such as prostaglandins (PGs) and interferon tau (IFNT), which are derived from both the conceptus and endometrium. These regulatory processes lead to specific alterations in the composition of ULF, which support conceptus development and implantation [7,10,11]. Supplementation with exogenous progesterone during days 12 to 14 of the estrous cycle alters ULF composition and promotes a uterine environment favorable for embryo development [6,10,12]. However, circulating P_4_ concentrations remain normal in dairy cows, whereas PG levels in ULF are reduced [13,14]. Given the established roles of PGs in regulating conceptus elongation and mediating endometrial responses to conceptus-derived signals [11,15,16,17], these findings suggest that PG signaling within the uterine environment may contribute to early pregnancy failure. Consistent with this hypothesis, inhibition of prostaglandin synthesis by intrauterine infusion of meloxicam, a selective inhibitor of prostaglandin-endoperoxide synthase 2 (PTGS2), suppresses prostaglandin production and alters ULF composition in dairy heifers during mid-diestrus [16,17]. In addition, PG concentrations in uterine fluid and the expression of prostaglandin-related genes in endometrium are closely associated with conceptus development and the maintenance of pregnancy [14,18]. However, the alterations of ULF compositions induced by PG in dairy cows remain unclear.

In the bovine uterine lumen, four principal bioactive prostaglandins have been identified, including prostaglandin E_2_ (PGE_2_), prostaglandin F_2α_ (PGF_2α_), 6-keto-prostaglandin F_1α_ (6-keto-PGF_1α_; a stable metabolite of prostacyclin, PGI_2_), and prostaglandin D_2_ (PGD_2_) [19]. Notably, PGE_2_ is recognized as a key immunomodulator and luteoprotective factor in ruminants, and its concentration in ULF is positively associated with conceptus length in dairy cows [14,20]. PGE_2_ levels in ULF increase markedly between days 12 and 18 of pregnancy, as well as during the corresponding period of the estrous cycle in dairy heifers [19]. During early pregnancy of cattle, PGE_2_ is primarily derived from the conceptus and endometrium, and the synthesis and secretion level in the elongating conceptuses are higher than the underlying endometrium [20,21,22]. PGE_2_ exerts diverse physiological effects through binding to E-type prostanoid (EP) receptors, including EP1, EP2, EP3, and EP4 [23]. In the bovine uterus, EP2 and EP4 are the predominant receptors, which are expressed in a temporal- and cell type–specific manner within the endometrium [24]. Due to the high expression of EP2 and EP4 in the endometrium and low levels in the conceptus during the peri-implantation period, PGE_2_ is thought to act primarily on the maternal endometrium [19,25]. Moreover, intrauterine perfusion of EP2 and EP4 antagonists has been shown to impair conceptus growth and elongation in sheep between days 10 and 16 of pregnancy [26].

Nevertheless, the functional roles and underlying molecular mechanisms of PGE_2_ that regulate ULF composition during early pregnancy in dairy cows remain poorly understood. In cattle, days 12–15 of the estrous cycle correspond to the period of conceptus elongation and are used as a physiological model for investigating conceptus development [6,10,27]. Herein, intrauterine infusion of PGE_2_ in dairy heifers on days 12–14 of the estrous cycle was used to investigate the effects of PGE_2_ on histotrophic composition and endometrial function. This study may enhance our understanding of the mechanisms underlying conceptus development by PGE_2_ and potentially inform strategies to improve pregnancy establishment and reproductive performance in dairy cows.

## 2. Materials and Methods

### 2.1. Animals and Experimental Design

In this study, all experiments were conducted in accordance with the Guide for the Care and Use of Agricultural Animals in Agricultural Research and Teaching and received approval from the Ethics Committee on the Use and Care of Animals at Northwest A&F University (Ethical Approval No. 2021100903).

Holstein dairy heifers (12 ± 2 months of age; body weight (BW): 360 ± 30 kg; body condition score (BCS): 3.0 ± 0.25) were housed in an open field and fed a total mixed ration (TMR) formulated for young cows once daily. All heifers were subjected to an estrous cycle synchronization program as previously described [16,17,28]. The experimental design and procedures are illustrated in Figure S1A. Briefly, estrous synchronization was initiated by administering PGF_2α_ (NSHF Ningbo Second Hormone Factory, Cixi, Zhejiang, China) on day −18, followed by gonadotropin-releasing hormone (GnRH) (NSHF Ningbo Second Hormone Factory, Cixi, Zhejiang, China) injections on day −15, −8, and 0. Additional PGF_2α_ injections were administered on days −3 and −2. The final GnRH administration was designated as day 0 of the estrous cycle. On this day, heifers were inseminated with sperm-free seminal plasma. Ovarian structures, including the corpus luteum (CL) and follicles, were examined by transrectal ultrasonography using a B-mode ultrasound scanner equipped with a 7.5 MHz linear-array probe (IMV Technologies Group, L’Aigle, France) on days 0 and 7 of the estrous cycle to assess synchronization of the estrous cycle in dairy heifers. Heifers that exhibited a dominant follicle and no CLs on day 0 and subsequently developed a CL on the same ovary by day 7 (Figure S1 and Table S1) were selected for further experimentation.

Dairy heifers with successful estrous synchronization (*n* = 12) were randomly assigned to two groups (Figure S1A). On days 12, 13, and 14 of the estrous cycle, heifers received an intrauterine infusion of either the PGE_2_ solution (*n* = 6) or vehicle (*n* = 6). The infusion was administered into the lumen of the uterine horn ipsilateral to the CL. One milligram of PGE_2_ (Cayman, Ann Arbor, MI, USA) was dissolved in 300 uL of dimethyl sulfoxide (DMSO) and then diluted with 5 mL of PBS. The vehicle consisted of the same preparation without PGE_2_. The selected dose of PGE_2_ was based on a previous report [29].

### 2.2. Collection of Uterine Lumen Fluid and Blood

On day 15 of the estrous cycle, each heifer received an injection of a 2% lidocaine hydrochloride (HCl) solution (Sichuan Jixing Animal Pharmaceutical Co., Ltd., Zigong, Sichuan, China) into the first coccygeal intervertebral space. Uterine lumen fluid was then collected from the uterine horn ipsilateral to CL, as previously described [17]. Briefly, the uterine horn was flushed via transcervical catheterization using 30 mL of PBS, and the recovered ULF was collected into a sterile tube. Samples with a recovered volume exceeding 15 mL and free from visible blood contamination were centrifuged at 4 °C and 2000× *g* for 20 min.

Blood samples were collected via puncture of the coccygeal blood vessels using Vacutainer tubes containing dipotassium ethylene diamine tetraacetic acid (EDTA) on days 0, 2, 4, 6, 8, 10, 12, 13, 14, and 15 of the estrous cycle. On days 12, 13, and 14, blood samples were collected after intrauterine perfusion for 1 h. Samples were immediately placed on ice and subsequently centrifuged at 3000× *g* for 10 min at 4 °C to isolate plasma. All samples were then divided equally and stored at −80 °C.

### 2.3. Detection of Plasma Progesterone Concentrations

The concentration of P_4_ in the blood plasma sample was measured using an Enzyme-Linked Immunosorbent Assay (ELISA) (002401, Jiangsu MEIMIAN Inc., Yancheng, China). The standard curve ranged from 0.1 to 100 ng/mL with a sensitivity of 0.15 ng/mL. Intra- and inter-assay coefficients of variation (CVs) were 8.7 and 11%, respectively.

### 2.4. Proteomic Sequencing Process and Analysis of ULF

Each treatment group included three biological replicates, with each replicate consisting of an equal volume of ULF collected from two dairy heifers. Proteomic sequencing and analysis methods were performed according to the previously described protocol [16], including protein extraction, peptide preparation, spectrogram database establishment, data-independent acquisition (DIA) mass spectrometry, and downstream bioinformatic analyses. Briefly, peptides were dissolved in a mobile phase A (0.1% formic acid, 2% acetonitrile/in water) and separated using an EASY-nLC 1200 ultra-high performance liquid chromatography (UHPLC) system (#LC140, Thermo Fisher, Waltham, MA, USA). Peptide separation was performed using a linear gradient of solvent B (0.1% formic acid in 90% acetonitrile) as follows: 5–25% over 60 min, 25–35% in 22 min, 35–80% in 4 min, and maintained at 80% for the final 4 min. Eluted peptides were ionized and analyzed using an Orbitrap Exploris 480 mass spectrometer (Thermo Fisher Scientific). Data acquisition was conducted in DIA mode. Raw DIA data were processed using the corresponding analysis software following standard workflows, including spectral library matching, peptide identification, and protein quantification. False discovery rate (FDR) control for peptide and protein identification was set at <1%. Protein intensity values were normalized prior to statistical analysis to minimize technical variation. DAPs were identified based on a fold change (FC) ≥1.5 or ≤0.67 with a *p*-value < 0.05. Functional annotation of DAPs was performed using Gene Ontology (GO) and Kyoto Encyclopedia of Genes and Genomes (KEGG) pathway analyses. Pathway enrichment was carried out using Gene Set Enrichment Analyses (GSEA) following the methodology described in the previous report [30]. Protein–protein interaction networks (PPIs) were constructed using the STRING dataset (version 11), and network visualization and analysis were performed using Cytoscape software (version 3.9.1).

### 2.5. Metabolome Extraction and Untargeted Metabolomics Analysis

Each group consisted of six replicates, with each sample derived from an individual dairy heifer. Untargeted metabolomic sequencing and data analysis were performed according to previous methods [17], including metabolite extraction, liquid chromatography-tandem mass spectrometry (LC–MS/MS) analysis, database search, and bioinformatic analysis. Metabolite extraction and LC–MS/MS analysis were conducted following the previous procedure [31]. Briefly, ULF samples were thawed on ice and mixed with a fourfold volume of extraction buffer (MeOH/ACN = 1:1, *v*/*v*). After vortex mixing and ultrasonic lysis, samples were incubated at −20 °C for 1 h to precipitate proteins, then centrifuged to collect the supernatant. The resulting extracts were dried and subsequently reconstituted in an equal volume of CAN:H_2_O (1:1, *v*/*v*), followed by centrifugation. The supernatant was transferred to a new tube for LC–MS analysis. Metabolite separation was performed using a Waters ACQUITY UPLC ultra-high-performance liquid phase system, and analytes were ionized using a capillary electrospray ion source and analyzed on a timesTOF Pro mass spectrometry system. Raw LC–MS data were processed using MetaboScape software (version 2022) for peak detection, retention time alignment, and signal normalization. Metabolite identification was performed by matching accurate mass and fragmentation spectra with reference databases. Differentially altered metabolites between the CON and PGE_2_ groups were screened based on the following criteria: variable importance in projection (VIP) ≥1, *p*-value < 0.05, and FC value ≥ 1.5 or FC ≤ 0.667. Multivariate statistical analysis was performed using orthogonal partial least squares–discriminant analysis (OPLS-DA) to evaluate metabolic differences between groups. Functional enrichment analysis of differential metabolites was performed using KEGG and Metabolite Set Enrichment Analysis (MSEA) to identify significantly enriched metabolic pathways.

### 2.6. Lipid Extraction and Targeted Lipidomic

Each group consisted of three replicates, with each replicate containing an equal volume of ULF from two dairy heifers. Lipidomic analysis was performed according to the report [16]. Briefly, lipids were extracted using the Methyl-tert-Butyl Ether (MTBE) extraction method. Lipid analyses were performed using a UHPLC Nexera LC-30A ultra-performance liquid chromatography system (SHIMADZU, Kyoto, Japan) coupled with a Q-Exactive Plus mass spectrometer (Thermo Scientific, Waltham, MA, USA). Raw mass spectrometry data were processed using the LipidSearch software (version 4.2, Thermo Scientific TM, Waltham, MA, USA) for lipid identification and quantification, including peak alignment, retention time correction, and extraction of the peak area. The resulting lipid intensity data were normalized using Perato scaling prior to statistical analysis. Multivariate statistical analysis was conducted using OPLS-DA to evaluate lipidomic differences between groups. Differentially altered lipids between the CON and PGE_2_ groups were identified based on the following criteria: VIP ≥ 1, FC ≥ 1.5 or ≤ 0.67, and *p*-value < 0.05. Functional enrichment analysis of differential lipids was performed using KEGG pathway analyses to identify significantly enriched lipid metabolic pathways.

### 2.7. Cell Culture and Treatment

Bovine endometrial epithelial cells (bEECs) were isolated from healthy dairy cows during the diestrus stage according to a previous report [32]. The bEECs were cultured in DMEM/F-12 medium supplemented with 10% fetal bovine serum (FBS) (Corning, NY, USA) and incubated at 37 °C in a humidified incubator with 5% CO_2_. When bEECs reached 50–60% confluence, the culture medium was replaced with phenol red-free DMEM/F-12 supplemented with 0.1% bovine serum albumin (BSA) (R&D Systems, Inc., Minneapolis, MN, USA) and incubated for 12 h for serum starvation. Subsequently, the bEECs were treated with phenol red-free DMEM/F-12 containing 10% FBS and P_4_ (10^−7^ M, Sigma, St. Louis, MO, USA) for 24 h. After P_4_ pretreatment, PGE_2_ (10^−8^ M, 10^−7^ M, 10^−6^ M; Cayman Chemical Company, Ann Arbor, MI, USA) was added to the culture medium for an additional 24 h. For IFNT treatment, cells were treated with 20 ng IFNT (C600063, Sangon Biotech Co., Ltd., Shanghai, China) for 12 h.

### 2.8. Scanning Electron Microscope

Cell samples were fixed in a 3% glutaraldehyde-buffered solution for 30 min at room temperature. The samples were then rinsed three times with phosphate-buffered solution (PBS) and post-fixed in 1% rhodium acid solution for 1 h at 4 °C. After washing with demineralized water, the samples were dehydrated through a graded ethanol series (30%, 50%, 70%, 90%, and 100%). Subsequently, the samples were critical-point dried using liquid CO_2_ (Leica EM CPD 300 Manual, Leica Microsystems, Wetzlar, Germany) and sputter-coated with a 7 nm gold/palladium layer using a Leica EM ACE 200 sputter coater. All samples were examined using a JSM-IT700HR scanning electron microscope (JEOL Ltd., Tokyo, Japan).

### 2.9. Western Blot

Cell samples were lysed with RIPA buffer (R0010, Solarbio, Beijing, China) following the manufacturer’s protocol. Protein concentrations were determined using the BCA protein assay kit (Nanjing Keygen Biotech Co., Ltd., Nanjing, China). Equal amounts of total proteins (20 μg) were separated by SDS-PAGE gel and subsequently transferred onto PVDF membranes (Millipore, Burlington, MA, USA). Membranes were blocked with 5% non-fat milk for 2 h at room temperature. The membranes were then incubated overnight at 4 °C with the following primary antibodies: anti-OPN antibody (1:1000, 22952-1-AP, Proteintech, Wuhan, China), anti-FN1 antibody (1:1000, 22952-1-AP, Proteintech), anti-ZO-1 antibody (1:1000, BY9025, Abways, Shanghai, China), anti-CDH1 antibody (1:1000, 3195S, Cell Signaling Technology, Danvers, MA, USA), anti-β-catenin antibody (1:500, sc-53484, Santa Cruz, CA, USA), anti-PTGER2 antibody (1:1000, ab167171, Abcam, Cambridge, UK), anti-STAT1 antibody (1:1000, 14994, Cell Signaling Technology), anti-p-STAT1 antibody (1:1000, 7649, Cell Signaling Technology), anti-IFNAR1 antibody (1:1000, A0575, ABclonal, Wuhan, China), anti-PTGER4 antibody (1:1000, ab217966, Abcam), and anti-β-Tubulin antibody (1:2000, 56739S, Cell signaling Technology). After washing three times with TBST, membranes were incubated with HRP-conjugated secondary antibody for 2 h at room temperature. Protein bands were detected using enhanced chemiluminescence (ECL) reagents. Protein expression levels were quantified using ImageJ software (version 1.48v).

### 2.10. RNA Extraction and Real-Time Quantitative PCR

Total RNA was extracted from the bEECs using TRIzol reagent (Takara, Tokyo, Japan) according to the manufacturer’s instructions. RNA concentration and purity were determined spectrophotometrically, and total RNA (1 μg) was reverse-transcribed into complementary DNA (cDNA) using the PrimeScript RT Master Mix reverse transcription kit (RR036, Takara). Real-time quantitative PCR (qPCR) was performed using SYBR Green Master Mix (Q311, Vazyme, Nanjing, China) on a Bio-Rad CFX96 system (Bio-Rad Laboratories, Inc., Hercules, CA, USA) following the manufacturer’s protocol. Relative gene expression levels were normalized to RPS9 as an internal reference gene and calculated using the 2^−ΔΔCt^ method. The primer sequences used in this study are listed in Supplementary Table S2.

### 2.11. Cell Transfection

For gene knockdown experiments, short hairpin RNAs (shRNAs) targeting PTGER2 or PTGER4, along with a negative control (shN), were cloned into the pCD513B-U6 vector to generate recombinant lentiviral vectors. Lentivirus packaging and cell transfection were performed as previously described [33,34]. Stable knockdown cell lines were established through puromycin selection (ST551, Beyotime, Shanghai, China). The target sequences for shRNA were as follows: shPTGER2, GAGCCACTGTGGCTCCCCTCC and GGAGGGGAGCCACAGTGGCTC; shPTGER4, CTACACACTGGTATGCGGGCC and GGCCCGCATACCAGTGTGTAG; shN, TTCTCCGAACGTGTCACGT and ACGTGACACGTTCGGAGAA.

### 2.12. Statistical Analyses

Differential molecules in omics analyses were identified based on a combination of statistical significance (*p* < 0.05) and fold-change thresholds. To account for multiple testing, *p*-values were additionally adjusted using the Benjamini–Hochberg procedure to estimate the false discovery rate (FDR), and the corresponding FDR values are reported for reference.

The results are presented as the means ± standard error of the mean (SEM) obtained from at least three independent experiments. Statistical analyses were performed using GraphPad Prism software (version 8.0). Differences between the two groups were analyzed using unpaired Student’s *t*-tests, while comparisons among multiple groups were performed using one-way analysis of variance (ANOVA) followed by Tukey’s post hoc test. Statistically significant differences are denoted as *p* < 0.05 (*), *p* < 0.01 (**), and *p* > 0.05 (no significance, ns).

## 3. Results

### 3.1. Hormone Detection in Plasma After Intrauterine Infusion of PGE_2_ in Dairy Heifers

**Figure 1:**
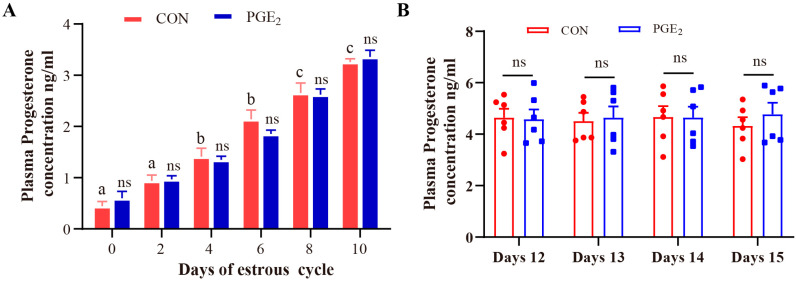
The difference in progesterone in plasma samples of dairy heifers between the CON and PGE_2_ groups. (**A**) Analysis of progesterone concentration in plasma samples of dairy heifers on different days before intrauterine perfusion of PGE_2_. (**B**) Analysis of progesterone concentration in plasma samples of dairy heifers on different days after intrauterine perfusion of PGE_2_. “ns” represents *p* > 0.05. Different letters above the bars indicate significant differences at *p* < 0.05, and the same letter indicates not significantly different (*p* > 0.05).

Transrectal ultrasonography (US) showed that the bilateral ovarian tissues of all dairy heifers lacked CLs on day 0 of the estrous cycle and exhibited a dominant follicle measuring approximately 1.7–2.3 cm. No significant difference was observed between the CON and PGE_2_ groups (*p* = 0.7872) (Figure S1B and Table S1). On day 7 of the estrous cycle, a CL—either homogenous (CL_hom_) or cavity-containing (CL_cav_)—was detected in one ovary of each heifer, with a size ranging from 2.4 to 3.2 cm. The luteal size in the PGE_2_ group did not differ significantly from that in the CON group (*p* = 0.9060) (Figure S1C and Table S1). Similarly, plasma P_4_ concentration showed no significant difference between the CON and PGE_2_ groups before intrauterine infusion, and P_4_ levels increased following CL formation (Figure 1A). After intrauterine infusion of PGE_2_, the plasma P_4_ concentration remained comparable between the CON and PGE_2_ groups on days 12, 13, 14, and 15 of the estrous cycle (Figure 1B). Consistent with these findings, US revealed that an intrauterine infusion of PGE_2_ had no significant effect on the size of CLs (*p* = 0.9733) and ovarian follicles in dairy heifers (*p* > 0.9999) (Figure S1D).

### 3.2. Proteomics Profiling of ULF in Dairy Heifers After Intrauterine Infusion of PGE_2_

**Figure 2:**
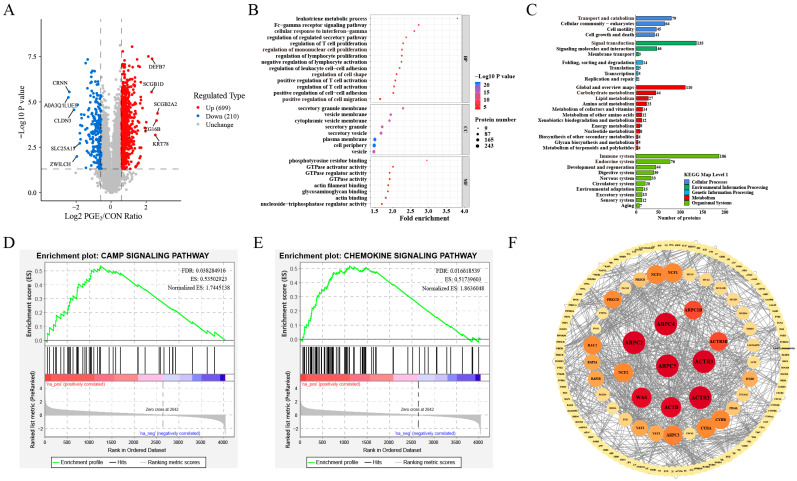
Proteomics analysis of ULF in dairy heifers between the CON and PGE_2_ groups. (**A**) Volcano plots of DAPs in ULF between the CON and PGE_2_ groups. (**B**) The bubble map of GO functional enrichment analysis of DAPs between the CON and PGE_2_ groups. (**C**) Pathway enrichment analysis of DAPs between the CON and PGE_2_ groups. (**D**,**E**) The GSEA analysis of cAMP and chemokine signaling pathways between the CON and PGE_2_ groups, respectively. (**F**) The protein–protein interaction network of DAPs based on MCC value between the CON and PGE_2_ groups. Nodes represent proteins and edges indicate protein–protein interactions. Node size reflects the degree of connectivity, with larger nodes representing proteins with higher interaction degrees.

Intrauterine infusion of PGE_2_ significantly altered the protein profile of ULF in *dairy* heifers. In total, 909 DAPs were identified between the CON and PGE_2_ groups, including 699 upregulated proteins and 210 downregulated proteins (Figure 2A; Table S3). GO analysis revealed that DAPs in the biological processes (BP) category were mainly associated with early embryo development (cell proliferation, cell migration, and regulation of embryonic cell shape), endometrial immunity responses (regulation of T-cell activation and cellular response to interferon-gamma), and cell adhesion processes (cell adhesion and adhesion junction organization), which were closely related to endometrial receptivity (Figure 2B; Table S4). In the cellular component (CC) terms, DAPs were primarily enriched in the secretory granule membrane and the vesicle membrane, whereas the molecular function (MF) category included phosphotyrosine residue binding, GTPase activator activity, and actin filament binding (Figure 2B). Further analysis showed that upregulated DAPs were mainly enriched in the cellular response to interferon-gamma, regulation of T-cell activation, mononuclear cell proliferation, regulation of cell shape, cell adhesion, and cell migration (Figure S2A). In contrast, downregulated DAPs were primarily associated with cholesterol, sterol, monocarboxylic acid, and the short-chain fatty acid biosynthetic process (Figure S2B). KEGG pathway analysis showed that DAPs were predominantly involved in pathways related to the immune and endocrine system, signal transduction, and transport and catabolism (Figure 2C, Table S5). Specifically, upregulated DAPs were mainly involved in leukocyte transendothelial migration, Fc gamma R-mediated phagocytosis, the cAMP signal pathway, the chemokine signaling pathway, the NOD-like receptor signaling pathway, the Rap1 signaling pathway, tight junctions, and focal adhesion (Figure S2C), while downregulated DAPs were enriched in metabolic pathways, including pyruvate metabolism, glutathione metabolism, glycolysis/gluconeogenesis, and steroid biosynthesis (Figure S2D). Notably, the cAMP signaling pathway (Figure 2D) and chemokine signaling pathway (Figure 2E) were enhanced following intrauterine PGE_2_ infusion. Moreover, PPI analysis identified ACTR2, ACTR3, ARPC2, ARPC4, and ARPC5 proteins as central hub proteins within the interaction network (Figure 2F). These proteins are primarily involved in cell migration, proliferation, and immune regulation.

### 3.3. Influence of PGE_2_ on Lipid Metabolism in ULF

**Figure 3:**
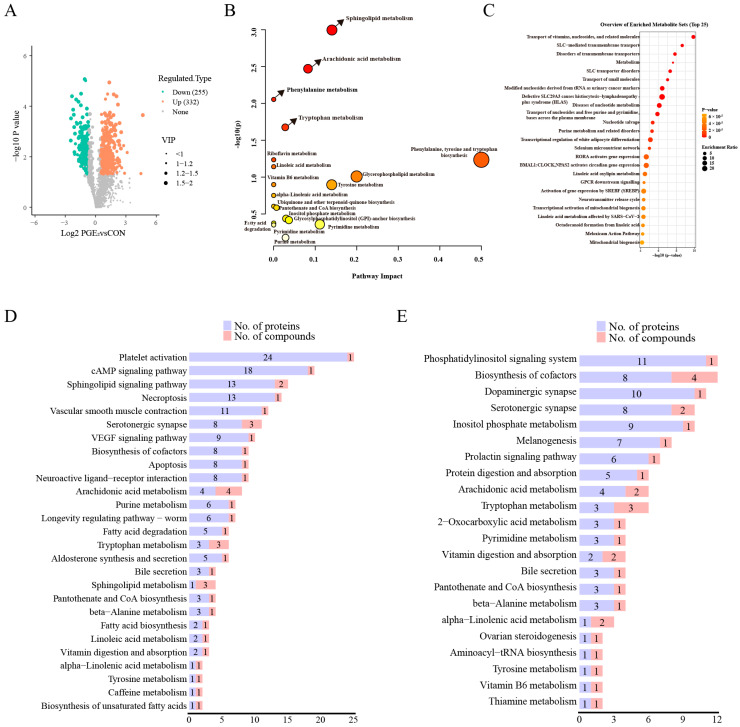
Metabolomic analysis of ULF in dairy heifers between the CON and PGE_2_ groups. (**A**) Volcano plots of differential metabolites in ULF between the CON and PGE_2_ groups. (**B**) The bubble map of differential metabolite pathway enrichment analysis between the CON and PGE_2_ groups. Circles represent metabolic pathways. Darker (redder) circles indicate greater changes in metabolites within each pathway, while circle size reflects the pathway impact score. Arrows indicate significantly altered pathways. (**C**) The bubble map of metabolite set enrichment analysis between the CON and PGE_2_ groups. (**D**) KEGG pathway mapping analysis using DAPs and differential metabolites from positive ion mode between the CON and PGE_2_ groups. (**E**) KEGG pathway mapping analysis using DAPs and differential metabolites from negative ion mode.

Because proteomic analysis revealed significant enrichment of metabolism-related pathways in ULF following intrauterine PGE_2_ infusion, we further investigated metabolic alterations in ULF. A total of 1911 metabolites were annotated in ULF samples (Table S6). The PCA score plots indicated an overall separation trend between the CON and PGE_2_ groups (Figure S3A). Further multivariate analysis using OPLS-DA clearly distinguished the two groups, with model parameters of R^2^Y = 0.946 and Q^2^ = 0.331 (Figure S3B), indicating good model fitness and predictive capability. Based on the screening criteria, 587 differentially altered metabolites were identified between the CON and PGE_2_ groups (Figure 3A), including 332 upregulated metabolites and 255 downregulated metabolites (Table S7). The top 20 upregulated and downregulated metabolites are presented in Figure S3D, including hippuric acid, n-benzylformamide, 2,8-Dihydroxyquinoline-beta-D-glucuronide, salicyluric acid, sphinganine, indoline, 4-(2-hydroxyethyl)piperazine-1-ethanesulfonic acid (HEPES), indolelactic acid, dl-phenylalanine, and dl-tryptophan. The differential metabolites were mainly categorized into lipids and lipid-like molecules (Figure S3E). Metabolic pathway enrichment analysis further demonstrated that the differential metabolites mainly focused on lipid metabolism and amino acid metabolism (Figure S3G), and were significantly enriched in sphingolipid metabolism, arachidonic acid metabolism, phenylalanine metabolism, and tryptophan metabolism (Figure 3B). To further explore functional differences between the CON and PGE_2_ groups, MSEA was performed based on untargeted metabolomics data. The results showed that differentially abundant metabolites were mainly involved in pathways related to vitamin and nucleoside transport, SLC-mediated transmembrane transport, and metabolic processes associated with transporter function (Figure 3C).

By integrating proteomics and metabolomics data, we further identified the key pathways responsive to PGE_2_. According to positive and negative ion modes, KEGG pathway analysis revealed that DAPs and differential metabolites were jointly enriched in the sphingolipid signaling pathway, arachidonic acid metabolism, α-linolenic acid metabolism, serotonergic synapse, biosynthesis of cofactors, and tryptophan metabolism. (Figure 3D,E). These pathways were mainly related to lipid metabolism.

**Figure 4:**
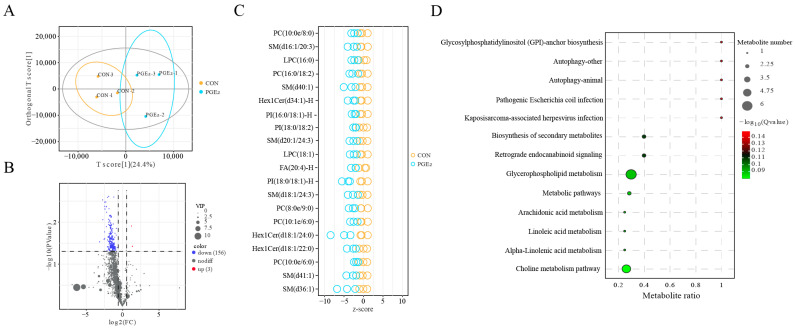
Lipidomic analysis of ULF in dairy heifers between the CON and PGE_2_ groups. (**A**) OPLS-DA score plots of lipidomic analysis in ULF between the CON and PGE_2_ groups. (**B**) Volcano plots of different lipid metabolites in ULF between the CON and PGE_2_ groups. (**C**) The top 20 differential lipid metabolites based on the VIP value between the CON and PGE_2_ groups. (**D**) Pathway enrichment analysis for differential lipid metabolites in ULF between the CON and PGE_2_ groups.

To further characterize the lipid alterations in ULF induced by PGE_2_, targeted lipidomic analysis of ULF was performed. The OPLS-DA results are shown in Figure 4A. A total of 1022 lipid metabolites belonging to 30 classes were detected in the positive ion mode, while 714 lipid metabolites belonging to 26 classes were detected in the negative mode (Table S8). Overall, 159 lipid metabolites were significantly altered (Figure 4B, Table S8). Among them, only Cer (m35:2+O), Cer (d18:1/17:1), and TG (8:0/8:0/10:0) were increased after PGE_2_ treatment, whereas the remaining 156 differentially altered lipids showed decreased concentrations (Table S9). The top 20 downregulated lipid metabolites based on VIP value are presented in Figure 4C. Pathway enrichment analysis indicated that these differentially altered lipids were mainly involved in glycerophospholipid metabolism, metabolic pathway, choline metabolism, biosynthesis of secondary metabolites, arachidonic acid metabolism, and retrograde endocannabinoid signaling (Figure 4D). Collectively, PGE_2_ treatment significantly altered the lipid profile of ULF and suppressed lipid accumulation in dairy heifers.

### 3.4. Integrated Proteomics and Lipidomics to Identify the Key Lipid Metabolic Pathways Induced by PGE_2_

**Figure 5:**
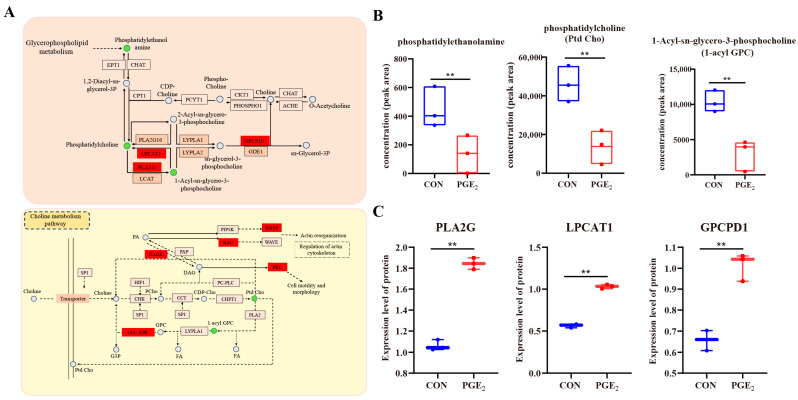
Comparison of proteome and lipidome profiles in ULF between the CON and PGE_2_ groups. (**A**) A map depicting the glycerophospholipid and choline metabolism pathways. Circles represent metabolites, with green circles indicating decreased abundance in uterine luminal fluid following PGE_2_ treatment. Rectangles represent proteins, with red and brown rectangles indicating upregulated and downregulated proteins, respectively. (**B**) Analysis of differentially altered metabolites in glycerophospholipid and choline metabolism pathways. (**C**) Analysis of protein expression involved in glycerophospholipid metabolism and choline metabolism in the CON and PGE_2_ groups. “**” represents *p* < 0.01.

Joint analysis of proteins and lipids could provide a more comprehensive understanding of lipid metabolic pathways and related physiological processes. Therefore, Spearman’s correlation analysis was performed between DAPs and differential lipid metabolites. The results showed that 292 protein–lipid pairs exhibited strong correlations between the CON and PGE_2_ groups (r ≥ 0.9 or r ≤ −0.9), including 66 positive correlations and 226 negative correlations (Figure S4A). In addition, 5867 protein–lipid pairs showed strong correlations at a slightly lower threshold (r ≥ 0.8 or r ≤ −0.8) between DAPs and differential lipid metabolites (Table S10). Among the differential lipids, Hex1Cer(d20:0/24:0), PI(18:0/18:2)-H, CerG3GNAc1(d44:1), Hex1Cer(d44:0+O), Hex1Cer(d44:2), and LPC(16:1e) interacted with the majority of DAPs (Figure S4B). Functional annotation revealed that these associated proteins were mainly involved in cell activation (Figure S4C). Specifically, both the differentially accumulated lipid metabolites and DAPs were mainly enriched in glycerophospholipid metabolism and the choline metabolism pathway. To further elucidate the interactions between DAPs and differential lipid metabolites, a metabolic pathway network was constructed (Figure 5A). In the glycerophospholipid metabolism pathway, phosphatidylethanolamine, phosphatidylcholine (Ptd Cho), and 1-Acyl-sn-glycero-3-phosphocholine (1-acyl GPC) were downregulated after PGE_2_ treatment (Figure 5B). Conversely, the abundance of PLA2, lysophosphatidylcholine acyltransferase (LPCAT1/2), and glycerophosphocholine phosphodiesterase (GPCPD1) increased after PGE_2_ treatment (Figure 5C). Ptd Cho and 1-acyl GPC were also involved in the pathway of choline metabolism pathway; however, the abundance of the key proteins in this pathway remained unchanged, except for GPCPD1. Collectively, these findings indicated that the proteins associated with cell activation were closely linked to lipid alterations induced by PGE_2_ treatment in dairy heifers.

### 3.5. PGE_2_ Modulates Adhesion-Related Proteins in bEECs

**Figure 6:**
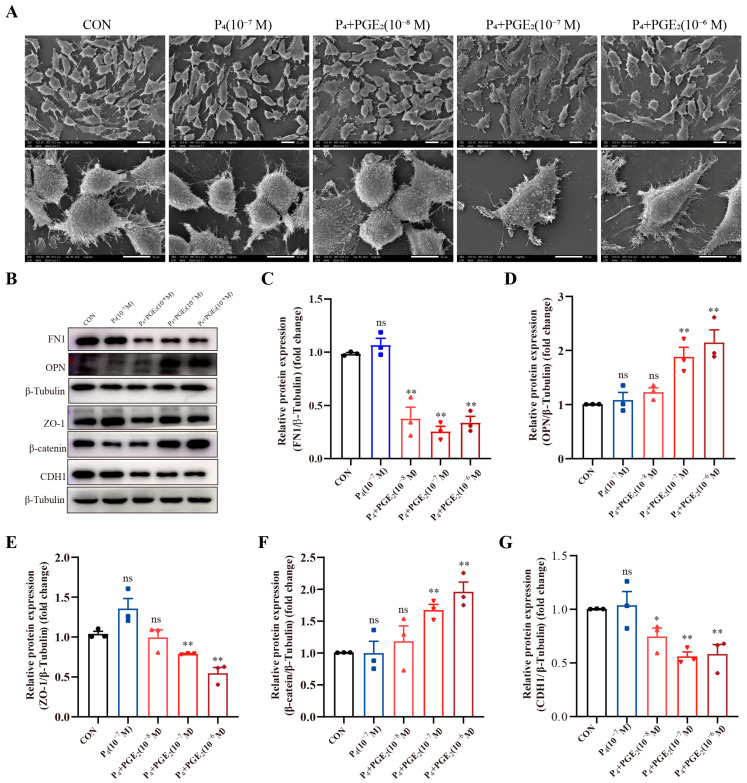
The effect of PGE_2_ on adhesive capacity in bovine endometrial epithelial cells. (**A**) Representative images of microvilli in bEECs after PGE_2_ treatment with different concentrations (10^−8^, 10^−7^, and 10^−6^ M). (**B**) Representative images of OPN, ZO-1, CDH1, FN1, and β-catenin protein expression in bEECs after PGE_2_ treatment with different concentrations (10^−8^, 10^−7^, and 10^−6^ M). (**C**–**G**). Analyses of FN1, OPN, ZO-1, β-catenin, and CDH1 protein expression in bEECs after PGE_2_ treatment with different concentrations (10^−8^, 10^−7^, and 10^−6^ M), respectively. “**” represents *p* < 0.01; “*” represents *p* < 0.05; “ns” represents *p* > 0.05.

The composition of ULF is mainly regulated by secretions from endometrial and epithelial cell secretions, and the biological effects of PGE_2_ are mainly exerted on the endometrium [6]. Proteomics analysis revealed that the DAPs were mainly associated with positive regulation of cell adhesion (Figure S5A,B), including pathways related to regulation of actin cytoskeleton (Figure S5C), cell adhesion molecules (Figure S5D), and focal adhesion (Figure S2E). To further validate the effect of PGE_2_ on endometrial function, in vitro experiments using bEECs were conducted. Scanning electron microscopy revealed that PGE_2_ treatment reduced the number of microvilli on the cell surface and induced cell flattening in bEECs. The most pronounced morphological changes were observed at a concentration of 10^−7^ M (Figure 6A). These structural alterations are closely related to cell remodeling and may influence the adhesive properties of bEECs. Subsequently, the expression levels of proteins involved in cell remodeling were examined under different concentrations of PGE_2_ treatment (Figure 6B), including the cell tight junction protein (ZO-1) and adhesion-related proteins (OPN, FN1, CDH1, and β-catenin). The results showed that the expression levels of FN1, CDH1, and ZO-1 were significantly downregulated after PGE_2_ treatment (Figure 6C,E,G), whereas OPN and β-catenin were significantly upregulated (Figure 6D,F). Collectively, these results suggest that PGE_2_ treatment may promote epithelial remodeling in bEECs and thereby influence endometrial adhesion processes.

### 3.6. PGE_2_ Modulates Interferon Tau Signaling in bEECs

**Figure 7:**
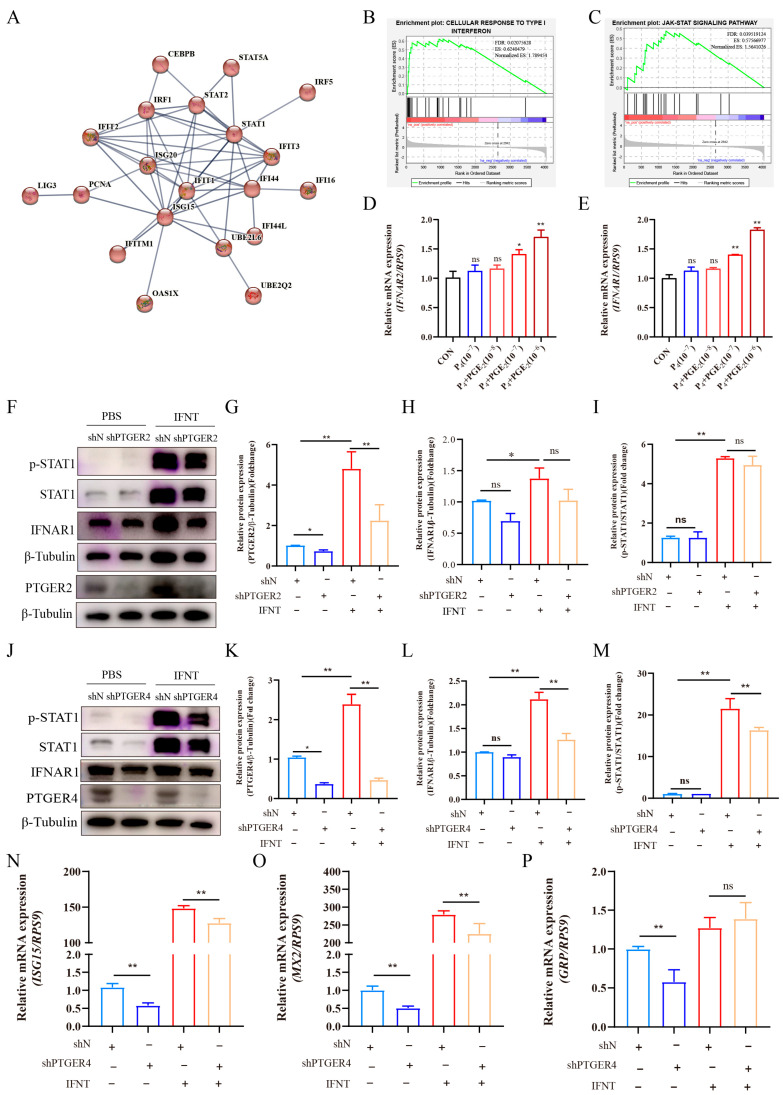
The effect of PGE_2_ treatment on IFNT receptor expression in bEEC. (**A**) PPI network analysis of DAPs involved in cellular response to type I interferon. (**B**) GSEA analysis of cellular response to the type I interferon pathway. (**C**) GSEA analysis of the JAK-STAT signaling pathway. (**D**) Analysis of *IFNAR1* expression in bEECs after PGE_2_ treatment with different concentrations (10^−8^, 10^−7^, and 10^−6^ M). (**E**) Analysis of *IFNAR2* expression in bEECs after PGE_2_ treatment with different concentrations (10^−8^, 10^−7^, and 10^−6^ M). (**F**) Representative images of PTGER2, IFNAR1, STAT1, and p-STAT1 protein expression in bEECs after interference with PTGER2. (**G**–**I**) Analyses of PTGER2, IFNAR1, and p-STAT1 protein expression in bEECs after interference with PTGER2, respectively. (**J**) Representative images of PTGER4, IFNAR1, STAT1, and p-STAT1 protein expression in bEECs after interference with PTGER4. (**K**–**M**) Analyses of PTGER4, IFNAR1, and p-STAT1 protein expression in bEECs after interference with PTGER4, respectively. (**N**–**P**) Analysis of *ISG15*, *MX2,* and *GRP* expression in bEECs after interference with PTGER4, respectively. “**” represents *p* < 0.01; “*” represents *p* < 0.05; “ns” represents *p* > 0.05.

According to the analysis of DAPs between the CON and PGE_2_ groups, PGE_2_ treatment was found to affect the response to type I interferon signaling (Figure 7A), including proteins such as ITG15, UBE2L6, STAT1, and others. Furthermore, GSEA revealed that PGE_2_ treatment significantly enhanced the cellular response to type I interferon (Figure 7B) and the JAK-STAT signaling pathway (Figure 7C). In ruminants, IFNT is a unique type I interferon and functions as the maternal recognition signal of pregnancy. IFNT regulates downstream classical and non-classical interferon-stimulated genes (ISGs) by binding to its receptors, IFNAR1 and IFNAR2. Therefore, the expression levels of *IFNAR1* and *IFNAR2* in bEECs were examined following treatment with different concentrations of PGE_2_. As the concentration of PGE_2_ increased, the expression levels of *IFNAR1* and *IFNAR2* were correspondingly elevated (Figure 7D,E). These findings suggest that PGE_2_ may influence the responsiveness of bEECs to IFNT by regulating the expression of IFNT receptors.

PGE_2_ mainly exerts its biological effects by binding to its receptors, PTGER2 and PTGER4. To further investigate the roles of PGE_2_ in IFNT signaling, stable bEEC cell lines with PTGER2 or PTGER4 knockdown were constructed. When PTGER2 expression was suppressed in bEECs (Figure 7F,G), neither IFNAR1 expression (Figure 7H) nor the p-STAT1/STAT1 ratio (Figure 7I) showed significant changes under IFNT stimulation. Conversely, knockdown of PTGER4 significantly reduced IFNAR1 expression in bEECs in the presence of IFNT (Figure 7J–L). Compared with the shN control group, the p-STAT1/STAT1 ratio was also significantly decreased in the shPTGER4 group (Figure 7M). Furthermore, the expression levels of classical interferon-stimulated genes (*ISG15* and *MX2*) and the non-classical interferon-stimulated gene (*GRP*) were examined in bEECs. PTGER4 knockdown significantly reduced the expression of *ISG15*, *MX2,* and *GRP* (Figure 7N–P). Under IFNT stimulation, PTGER4 interference markedly decreased *ISG15* and *MX2* expression, whereas no significant change was observed in *GRP* expression. Collectively, these findings indicate that PGE_2_ primarily modulates the responsiveness of bEECs to IFNT through the PTGER4 receptor.

## 4. Discussion

In ruminants, the PGE_2_ concentration in ULF and the expression of prostaglandin-related genes in the endometrium are closely linked to fertility [13,14,18]. Therefore, a deeper understanding of how PGE_2_ regulates the uterine environment is essential for improving reproductive efficiency. In this study, an estrous cycle model was deliberately employed to isolate the direct effects of PGE_2_ on the endometrium and ULF in the absence of conceptus-derived signals. During early pregnancy, the uterine environment is simultaneously influenced by conceptus-secreted factors, particularly IFNT, which may confound the interpretation of PGE_2_-specific effects. Thus, intrauterine infusion of PGE_2_ during mid-diestrus provides a controlled physiological context in which the endometrial and histotrophic responses to PGE_2_ can be examined independently of conceptus-mediated regulation. Determining the precise physiological concentration of PGE_2_ in bovine uterine luminal fluid remains technically challenging. PGs are locally synthesized within the uterus, rapidly metabolized, and dynamically regulated by the stage of the estrous cycle or pregnancy, as well as the uterine microenvironment. Previous studies have reported that PGE_2_ concentrations in bovine uterine luminal fluid range from approximately 0.2 to 3 ng/mL [14,19]. However, most available measurements are obtained from uterine flushing samples collected after slaughter; therefore, the exact in vivo luminal concentrations remain difficult to determine. In this study, intrauterine infusion of 1 mg PGE_2_ was used to ensure biologically effective local stimulation of prostaglandin signaling during days 12–14 of the estrous cycle. Proteomics analysis showed that PGE_2_ treatment significantly upregulated the cAMP signaling pathway and chemokine signaling pathway, both of which are well-established downstream pathways activated by PGE_2_. During the pre-implantation period, PGE_2_ activates the cAMP signaling pathway to regulate pregnancy-related gene expression in bovine endometrial stromal cells [35]. In addition, PTGER2 has been reported to mediate growth factor gene expression through the activation of the cAMP signaling pathway in bovine endometrial epithelial cells [36]. These findings collectively indicate that the administered dose effectively activated PGE_2_-mediated signaling within the uterus. Nevertheless, direct quantification of uterine luminal PGE_2_ concentrations following intrauterine infusion would further strengthen the physiological interpretation of the treatment. Future studies incorporating real-time or in situ measurement of uterine prostaglandin levels may provide a more precise understanding of the local prostaglandin dynamics within the uterine microenvironment.

Days 12–15 of the estrous cycle are recognized as the window for initiation of conceptus elongation in cattle [6,10,12]. During this period, ULF undergoes a metabolic shift characterized by increasing levels of lipids essential for cell membrane formation, including phospholipids, phosphatidylethanolamine, and lysophospholipids, which play important roles in supporting conceptus elongation [6,12,37]. In ruminants, conceptus length and weight increase exponentially during the pre-implantation period, primarily as a result of rapid trophoblast cell proliferation [6]. Cell growth and proliferation require the synthesis of fundamental cellular components, including proteins, lipids, and nucleic acids. Consistently, the upregulation of genes related to lipid uptake and fatty acid synthesis indicates that rapidly proliferating trophoblast cells must obtain sufficient lipids from ULF [38]. Interestingly, PGE_2_ treatment resulted in a reduction in lipid accumulation within the uterine luminal fluid. Lipids constitute a critical component of the uterine microenvironment, functioning not only as metabolic substrates but also as signaling molecules, which support embryo development and facilitate maternal–embryo communication [14,39]. Therefore, alterations in luminal lipid levels likely reflect dynamic changes in uterine metabolic activity and the regulation of the local biochemical milieu. Cell-based validation experiments further indicated that prostaglandins may modulate the dynamic remodeling of endometrial epithelial cells. Collectively, we speculate that PGE_2_ may regulate the secretory activity of these cells, thereby reducing the release of lipid components into the uterine lumen. Alternatively, the observed decrease may reflect enhanced metabolic turnover or increased lipid utilization within the uterine environment. Notably, lipid droplets (LDs) in the endometrium are considered a major source of lipids present in the uterine luminal fluid in ruminants [6,10,28]. Building on our previous research demonstrating that PGE_2_ promotes LD accumulation in endometrial epithelial cells [34], we propose that PGE_2_ may facilitate intracellular lipid storage while simultaneously suppressing luminal secretion. Nevertheless, the precise molecular pathways underlying this process remain to be elucidated through further investigation.

**Figure 8:**
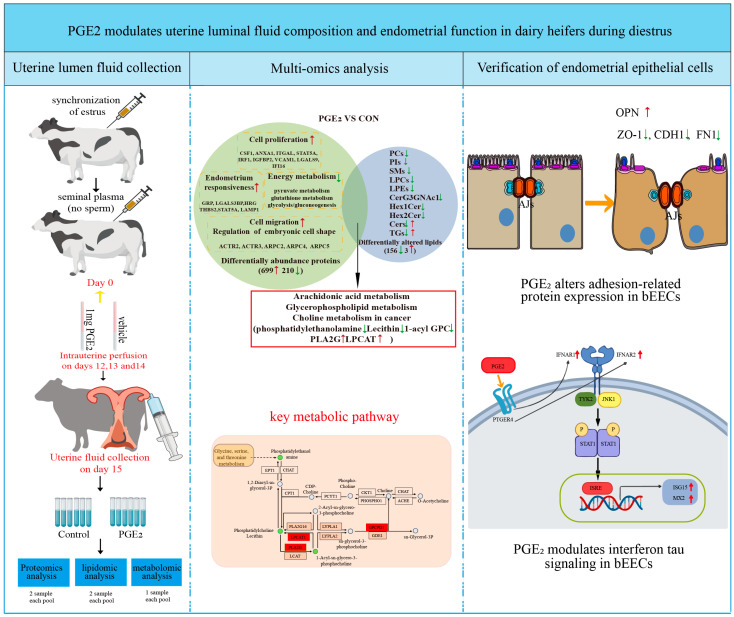
The schematic diagram of PGE_2_ treatment and alterations in uterine luminal fluid composition and endometrial function in dairy heifers.

Glycerophospholipid metabolism and choline metabolism are key metabolic pathways influenced by PGE_2_. Glycerophospholipids serve as fundamental components of cell membranes and act as precursors for bioactive lipid mediators, including icosanoids, lysophospholipids, and endocannabinoids [40]. Previous studies have reported that high-fertility heifers exhibit elevated levels of lipid metabolites in ULF compared with subfertile heifers, which are predominantly linked to glycerophospholipid metabolism and steroid biosynthesis [8]. In this study, the content of phosphatidylethanolamine, phosphatidylcholine lecithin, and l-acyl-cn-glycero-3-phosphocholine in ULF was significantly decreased following PGE_2_ treatment (Figure 8). These findings suggest that PGE_2_ negatively regulates glycerophospholipid metabolism within the uterine luminal environment. Consistent with these findings, inhibition of uterine prostaglandin production has been reported to disrupt physiological metabolic shifts by altering lipid composition of ULF in dairy heifers [16]. Moreover, the collective actions of multiple prostaglandins often have greater physiological relevance than the activity of a single prostaglandin or its concentration alone [19]. This observation further supports the concept that prostaglandins function within a coordinated regulatory network in the uterine lumen, where different prostaglandin species may interact to modulate metabolic processes and maintain the biochemical homeostasis of the uterine microenvironment.

In this study (Figure 8), the upregulated DAPs in the PGE_2_ group were mainly related to cell proliferation and cell migration. These results indicate that PGE_2_ may function as an important mediator in regulating the uterine environment to support conceptus cell proliferation and migration during the period of conceptus elongation. Notably, several members of the actin-related protein 2/3 (Arp2/3) complex, including ARPC4, ARPC2, ARPC5, ACTR3, and ACTR2, were significantly upregulated in ULF after PGE_2_ treatment. Consistent with this observation, the abundance of Arp2/3 complex subunits increases in ULF on day 16 of pregnancy compared with day 13 in cattle [41]. The Arp2/3 complex is a key regulator of actin cytoskeleton dynamics and facilitates trophoblast cell proliferation and motility by promoting F-actin polymerization and focal adhesion kinase (FAK) activation during early human pregnancy [42]. In addition, the Arp2/3 complex has been shown to regulate mouse embryo development by affecting cell division processes [43]. Collectively, these results suggested that PGE_2_ may modulate the uterine microenvironment in a manner that promotes trophoblast proliferation and migration.

Endometrial remodeling is a dynamic process that underlies both the estrous cycle and pregnancy establishment [44]. During implantation, the endometrial epithelium undergoes extensive structural and functional remodeling in response to pregnancy-related signals to establish uterine receptivity. Uterine receptivity is partially mediated by the distribution and functional capacity of microvilli on endometrial epithelial cells [45]. In cattle, the number of microvilli on the endometrial epithelium decreases between day 0 and day 14 of the estrous cycle [46]. This morphological change facilitates the formation of apical protrusions known as pinopodes, which are mainly induced by progesterone during the luteal phase and are associated with enhanced uterine secretory activity [45]. In this study, PGE_2_ treatment reduced the number of microvilli on the cell surface and induced a more flattened epithelial cell morphology. These morphological changes suggest that PGE_2_ may influence the formation of apical protrusions in the endometrial epithelium, reflecting epithelial remodeling that may affect both secretory activity and adhesion-related processes. The canonical Wnt/β-catenin pathway is a key regulator of cellular processes, such as cell proliferation, differentiation, invasion, and adhesion [47]. The interaction between CDH1 and β-catenin is essential for maintaining cell adhesion, and alterations in their localization and expression are closely associated with the initiation of implantation [48]. During early pregnancy in sheep and pigs, P_4_ stimulation downregulates the expression of uterine epithelial tight junction and adherens junction proteins, including ZO1 and CDH1 [49,50]. In this study, PGE_2_ treatment significantly decreased the expression of epithelial junction proteins (ZO-1 and CDH1) while increasing β-catenin expression in bEECs. Collectively, these findings suggest that PGE_2_ may participate in progesterone-mediated regulation of endometrial remodeling during the peri-implantation period.

GO analysis revealed that the upregulated DAPs in the PGE_2_ group were enriched in the cellular response to type I interferon, including IRF1, IRF5, ISG15, ISG20, IFI16, and STAT1. IFNT, classified as a type I interferon, functions as a unique pregnancy recognition signal secreted by the preimplantation conceptus in ruminants [51]. Endometrial responsiveness to IFNT is a critical determinant of fertility [52,53]. Previous studies have demonstrated that the expression of several IFNT-stimulated genes, including *ISG15, RSAD2, CST3, CTSL, GRP, LGALS15*, and *IGFBP1*, is significantly reduced in the endometrium when cyclic ewes receive intrauterine co-infusions of meloxicam and IFNT from days 10 to 14 post-estrus [15,54]. These findings suggest that prostaglandin signaling may play an important role in regulating the uterine response to IFNT. FNT exerts its biological effects by binding to a receptor complex composed of IFNAR1 and IFNAR2 subunits, thereby initiating downstream signaling cascades [51,55]. In this study, PGE_2_ treatment enhances the expression levels of IFNAR1 and IFNAR2 in bEECs. Furthermore, knockdown of PTGER4 resulted in a reduction in IFNAR1 expression in bEECs, indicating that PGE_2_ may regulate interferon receptor expression through PTGER4-mediated signaling. The downstream signaling pathway of type I interferons is well characterized and involves activation of tyrosine kinase 2 (TYK2), Janus kinase 1 (JAK1), and signal transducers and activators of transcription (STATs). Activation of the JAK/STAT pathway leads to STAT phosphorylation and the induction of ISGs [56]. Proteomics analysis of ULF demonstrated activation of the JAK-STAT1 pathway following PGE_2_ treatment. Consistently, silencing PTGER4 expression in bEECs reduced IFNT-induced STAT1 phosphorylation (the p-STAT1/STAT1 ratio) and significantly decreased the mRNA expression of classical IFNT-stimulated genes, including *ISG15* and *MX2.* Collectively, these results suggest that PGE_2_ may enhance endometrial responsiveness to IFNT by modulating the IFNAR–JAK–STAT signaling pathway (Figure 8), highlighting a potential role of PGE_2_ in coordinating endometrial signaling processes associated with early pregnancy. However, it should be noted that this study was conducted in non-pregnant animals. Therefore, the experimental model does not fully recapitulate the complex physiological environment of early pregnancy, particularly the presence of the conceptus and the dynamic signaling interactions between the conceptus and the maternal uterus. Future studies using pregnant animals will be necessary to further validate the physiological relevance of these findings for conceptus development and pregnancy establishment.

In this study, ULF samples from two animals were pooled for each replicate in proteomic and lipidomic analyses due to low biomolecule abundance in individual samples, which could affect the stability of downstream omics analyses. Although this strategy helped ensure sufficient material for reliable detection, pooling may mask inter-individual variation and potentially influence correlation analyses. Therefore, the results of this study should be interpreted primarily at the group level, and future studies using individual samples will be necessary to further validate these findings. Meanwhile, applying a strict FDR threshold substantially reduced the number of detectable molecules in this dataset. This is not unexpected in exploratory omics studies with moderate effect sizes and limited sample sizes. Therefore, the present study primarily used *p*-value and fold-change thresholds for initial screening.

## 5. Conclusions

PGE_2_ plays a pivotal role in regulating uterine function in dairy heifers during diestrus by inducing significant alterations in the proteomic and metabolomic profiles of uterine luminal fluid, with lipid metabolism emerging as the primary affected pathway. Furthermore, PGE_2_ modulates epithelial structure and adhesion-related protein expression and enhances the responsiveness of bovine endometrial epithelial cells to IFNT, primarily via the PTGER4 receptor. Collectively, these findings provide new insights into the molecular mechanisms underlying PGE_2_-mediated regulation of uterine function in dairy cattle.

## Data Availability

All data generated for this study are included in the article.

## Supplementary Materials

The following supporting information can be downloaded at https://www.mdpi.com/article/10.3390/ani16071037/s1. The supporting information was applied in Supplementary Materials (Figures S1–S5; Tables S1–S10). Figure S1: The experimental design of intrauterine perfusion of PGE_2_ and analysis of ovarian structure in dairy heifers. Figure S2: The enriched biological processes and pathways of DAPs between CON and PGE_2_ groups. Figure S3: The quality control of untargeted metabolomics sequence data between CON and PGE_2_ groups. Figure S4: The integration analysis both DAPs and differential lipid metabolites between CON and PGE_2_ groups. Figure S5: The analysis of DAPs between CON and PGE_2_ groups. Table S1: Ultrasonographic assessment of ovarian structures in heifers on different timepoints during the estrous cycles. Table S2: Primer pairs used for real time quantitative PCR. Table S3: The analysis of differentially abundance proteins (DAPs) between CON and PGE_2_ groups. Table S4: The GO terms (BP) of the DAPs between CON and PGE_2_ groups. Table S5: The KEGG analysis of the DAPs between CON and PGE_2_ groups. Table S6: All identified metabolites in positive and negative ion mode both CON and PGE_2_ groups. Table S7: List of differentially altered metabolites between CON and PGE_2_ groups. Table S8: The analysis of lipid metabolites in ULF both CON and PGE_2_ groups. Table S9: The analysis of the differentially altered lipids between CON and PGE_2_ groups. Table S10: The analysis of significantly correlated DAP-differential lipid metabolite pairs between CON and PGE_2_ groups.
