# Supplementary material for: PGE2 Modulates Uterine Luminal Fluid Composition and Endometrial Function in Dairy Heifers During Diestrus
Source: Animals (Basel). 2026 Mar 28;16(7):1037. doi: 10.3390/ani16071037 (PMC13072008; doi:10.3390/ani16071037)
Supplement: Supplementary file 1 [file animals-16-01037-s001.zip › animals-4193262-supplemental/animals-4193262-supplemental.pdf]

Supplementary materials

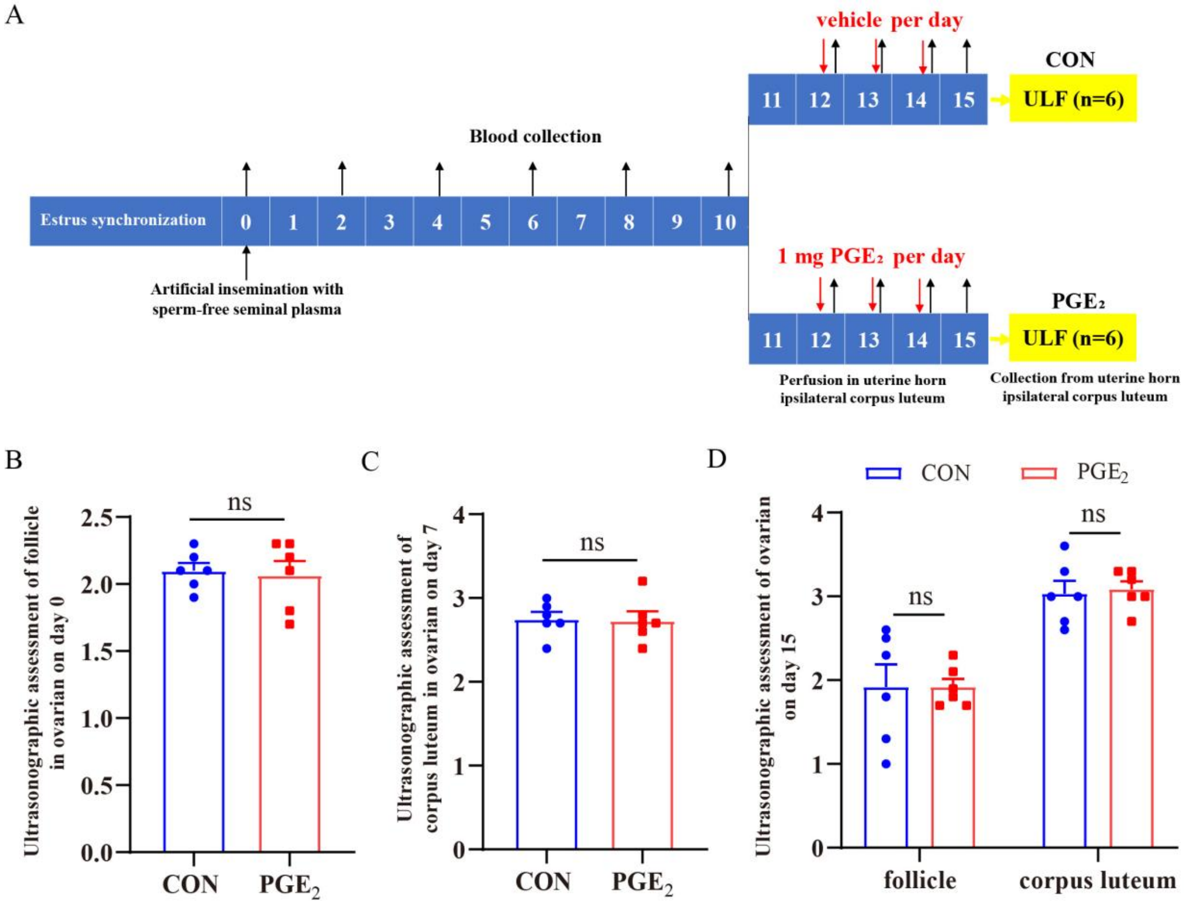

Figure S1. The experimental design of intrauterine perfusion of PGE_2 and analysis of ovarian structure in dairy heifers. A. The experimental design of intrauterine perfusion of PGE_2 in dairy heifers. B. Transrectal ultrasonography assessment of ovarian follicles on day 0 of estrous cycle using B-ultrasound. C. The analysis of corpus luteum (CL) in ovarian tissues on day 7 of estrous cycle. D. The analyses of follicles and CL in ovarian tissues on day 15 of estrous cycle; "ns" represent $P > 0.05$.

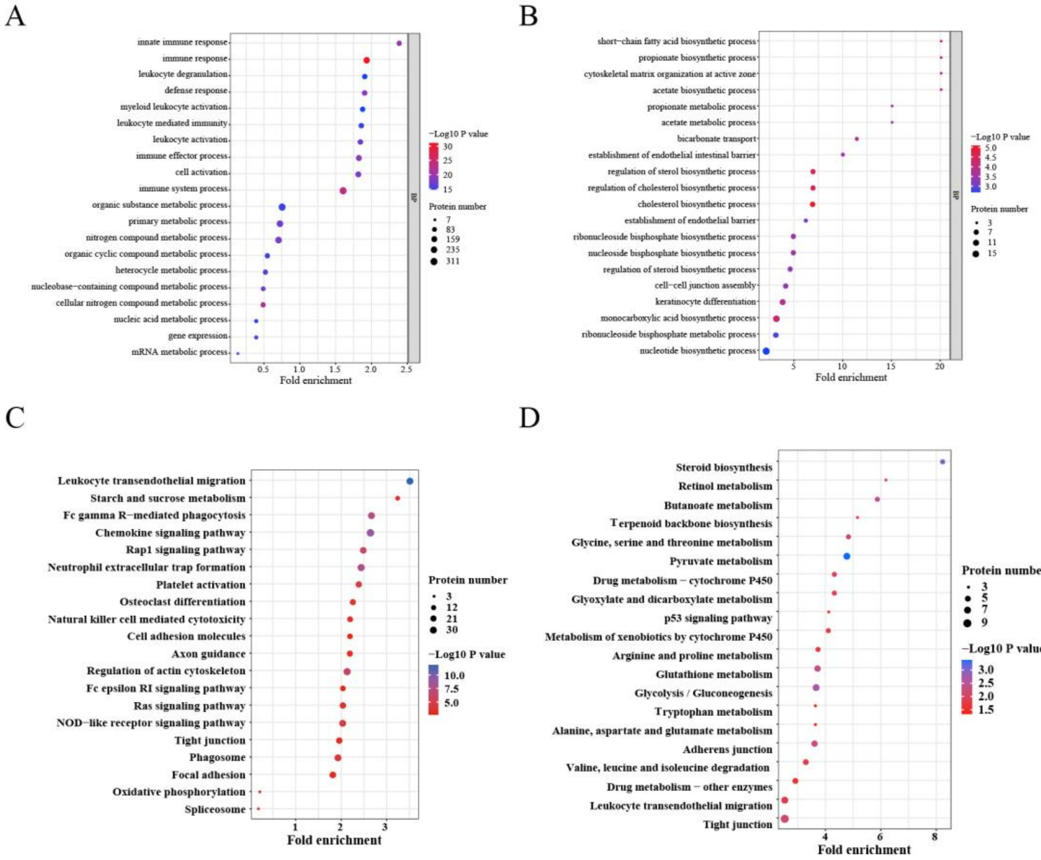

Figure S2. The enriched biological processes and pathways of DAPs between CON and PGE_2 groups. A. Top 20 GO terms (BP) of the up-regulated DAPs between CON and PGE_2 groups. B. Top 20 GO terms (BP) of the down-regulated DAPs between CON and PGE_2 groups. C. Top 20 pathways of the up-regulated DAPs between CON and PGE_2 groups. D. Top 20 pathways of the down-regulated DAPs between CON and PGE_2 groups.

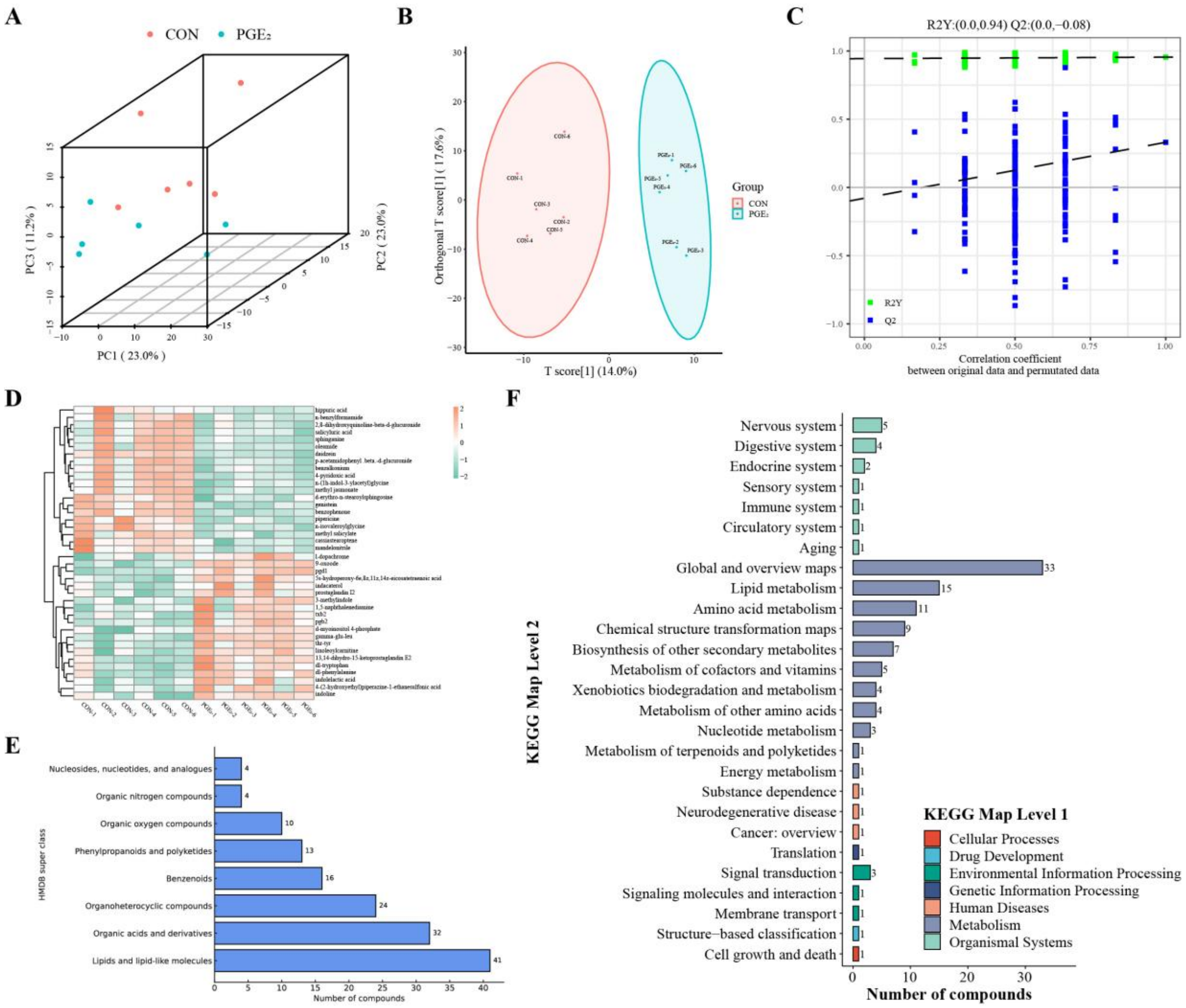

Figure S3. The quality control of untargeted metabolomics sequence data between CON and PGE_2 groups. A. The scattered plots of PCA analysis between CON and PGE_2 groups. B. The score plots of OPLS-DA between CON and PGE_2 groups. C. The permutation test plots between CON and PGE_2 group; Q^2=percentage of Y dispersions predicated by the model using cross-validation; R^2=percentage of Y dispersions explained by the model. D. The heatmap of top 20 up-regulated and down-regulated differential metabolites between CON and PGE_2 groups. E. Bar graph showing the classification of the differential metabolites. F. KEGG enrichment analysis of the differential metabolites.

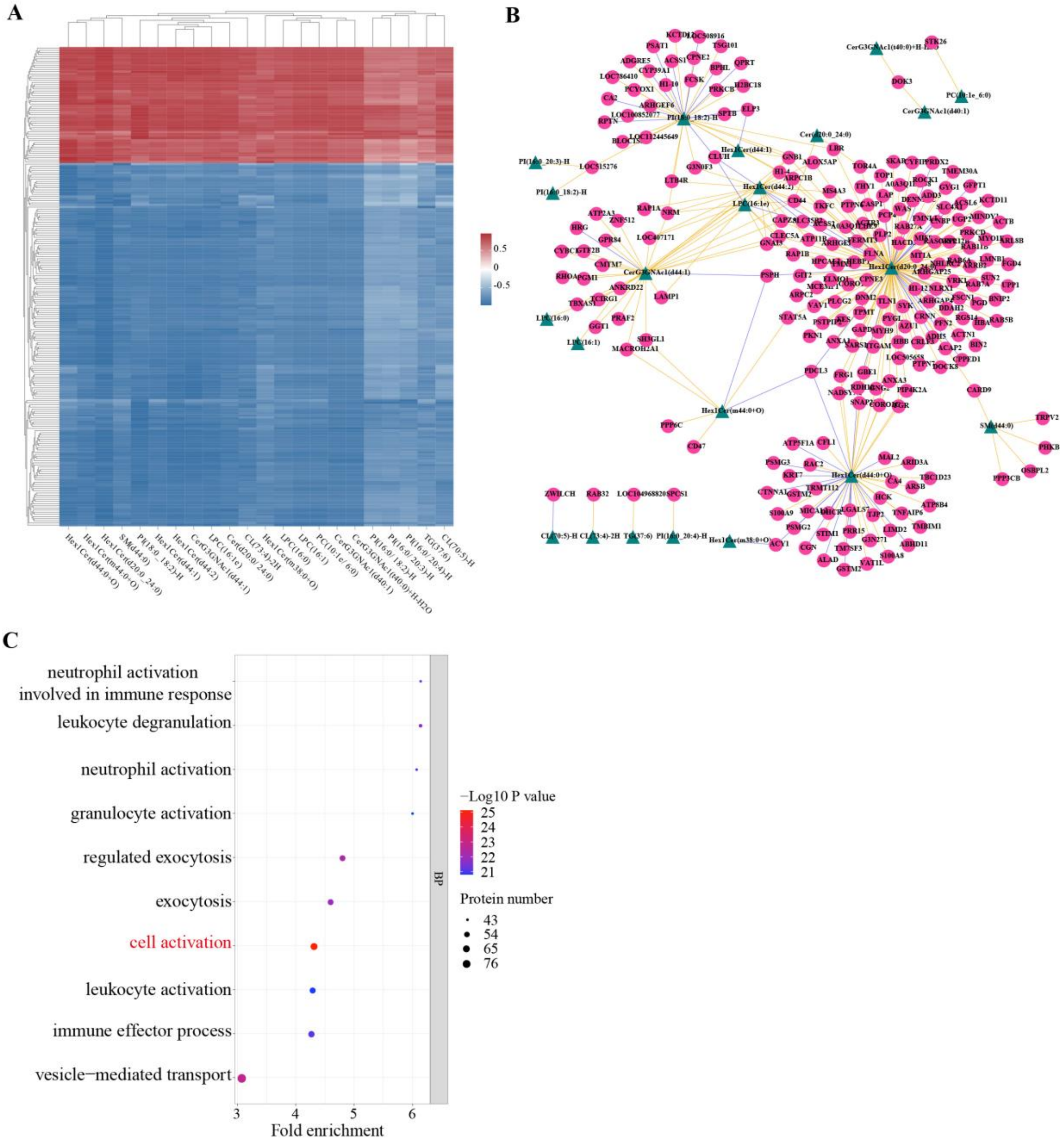

Figure S4. The integration analysis both DAPs and differential lipid metabolites between CON and PGE_2 groups. A. The heatmap of the strongly correlated DAPs and differential lipid metabolites between CON and PGE_2 groups (r≥0.9 or r≤-0.9). B. The correlation network of DAPs and differential lipid metabolites between CON and PGE_2 groups. C. The top 10 biological processes of DAPs strongly related to differential lipid metabolites between CON and PGE_2 groups.

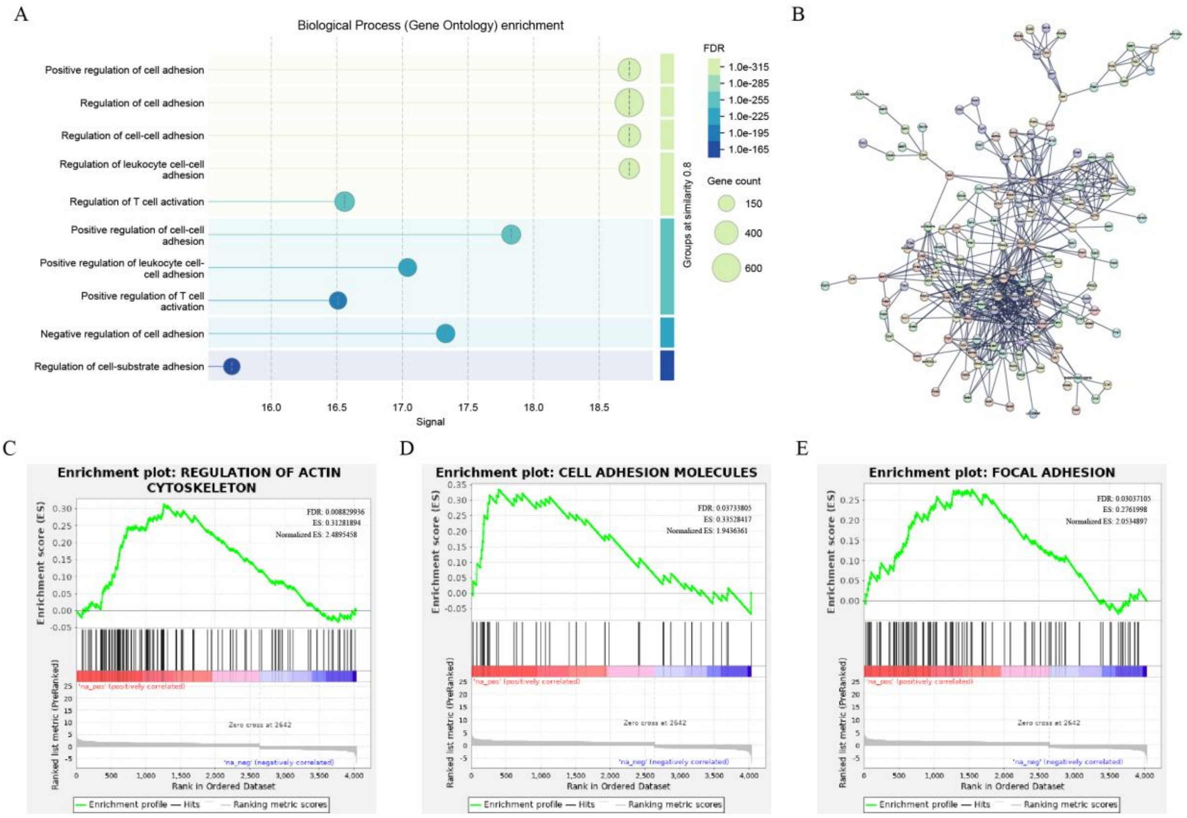

Figure S5. The analysis of DAPs between CON and PGE_2 groups. A. The BP classification of DAPs between CON and PGE_2 groups. B. The analyses of PPI with the DAPs based on highest confidence (0.900). C-E. The GSEA analysis of regulation of actin cytoskeleton, cell adhesion molecules, and focal adhesion signaling pathways between CON and PGE_2 groups, respectively.

Table legends

Table S1. Ultrasonographic assessment of ovarian structures in heifers on different timepoints during the estrous cycles

Table S2. Primer pairs used for real time quantitative PCR

Table S3. The analysis of differentially abundance proteins (DAPs) between CON and PGE_2 groups

Table S4. The GO terms (BP) of the DAPs between CON and PGE_2 groups

Table S5. The KEGG analysis of the DAPs between CON and PGE_2 groups

Table S6. All identified metabolites in positive and negative ion mode both CON and PGE_2 groups

Table S7. List of differentially altered metabolites between CON and PGE_2 groups

Table S8. The analysis of lipid metabolites in ULF both CON and PGE_2 groups

Table S9. The analysis of the differentially altered lipids between CON and PGE_2 groups

Table S10. The analysis of significantly correlated DAP-differential lipid metabolite pairs between CON and PGE_2 groups
